# Measurement of Epstein-Barr virus DNA load using a novel quantification standard containing two EBV DNA targets and SYBR Green I dye

**DOI:** 10.1186/1743-422X-7-252

**Published:** 2010-09-22

**Authors:** Meav-Lang J Lay, Robyn M Lucas, Mala Ratnamohan, Janette Taylor, Anne-Louise Ponsonby, Dominic E Dwyer

**Affiliations:** 1 Virology Department, Centre For Infectious Diseases & Microbiology Laboratory Services, Institute of Clinical Pathology & Medical Research, Institute Road, Westmead Hospital, Westmead 2145, New South Wales, Australia; 2 National Centre for Epidemiology and Population Health, The Australian National University, Canberra, ACT, 0200 Australia; 3 Murdoch Childrens Research Institute, 9th Floor AP Building, The Royal Children's Hospital, Flemington Road, Parkville, Victoria 3052, Australia

## Abstract

**Background:**

Reactivation of Epstein-Barr virus (EBV) infection may cause serious, life-threatening complications in immunocompromised individuals. EBV DNA is often detected in EBV-associated disease states, with viral load believed to be a reflection of virus activity. Two separate real-time quantitative polymerase chain reaction (QPCR) assays using SYBR Green I dye and a single quantification standard containing two EBV genes, Epstein-Barr nuclear antigen-1 (EBNA-1) and BamHI fragment H rightward open reading frame-1 (BHRF-1), were developed to detect and measure absolute EBV DNA load in patients with various EBV-associated diseases. EBV DNA loads and viral capsid antigen (VCA) IgG antibody titres were also quantified on a population sample.

**Results:**

EBV DNA was measurable in ethylenediaminetetraacetic acid (EDTA) whole blood, peripheral blood mononuclear cells (PBMCs), plasma and cerebrospinal fluid (CSF) samples. EBV DNA loads were detectable from 8.0 × 10^2 ^to 1.3 × 10^8 ^copies/ml in post-transplant lymphoproliferative disease (n = 5), 1.5 × 10^3 ^to 2.0 × 10^5 ^copies/ml in infectious mononucleosis (n = 7), 7.5 × 10^4 ^to 1.1 × 10^5 ^copies/ml in EBV-associated haemophagocytic syndrome (n = 1), 2.0 × 10^2 ^to 5.6 × 10^3 ^copies/ml in HIV-infected patients (n = 12), and 2.0 × 10^2 ^to 9.1 × 10^4 ^copies/ml in the population sample (n = 218). EBNA-1 and BHRF-1 DNA were detected in 11.0% and 21.6% of the population sample respectively. There was a modest correlation between VCA IgG antibody titre and BHRF-1 DNA load (rho = 0.13, p = 0.05) but not EBNA-1 DNA load (rho = 0.11, p = 0.11).

**Conclusion:**

Two sensitive and specific real-time PCR assays using SYBR Green I dye and a single quantification standard containing two EBV DNA targets, were developed for the detection and measurement of EBV DNA load in a variety of clinical samples. These assays have application in the investigation of EBV-related illnesses in immunocompromised individuals.

## Background

Epstein-Barr virus (EBV) causes infectious mononucleosis, an acute but self-limiting disease affecting children and young adults. After primary infection, the virus persists indefinitely in B-lymphocytes [1], only to reactivate when cellular immunity is impaired. In immunocompromised individuals, EBV-related disorders following virus reactivation are associated with significant morbidity and mortality [2]. Up to 15% of transplant recipients develop post-transplant lymphoproliferative disease (PTLD), a heterogeneous group of disorders characterised by EBV transformation of lymphocytes [3,4]. Although uncommon, PTLD is aggressive and coupled with high mortality rates of 50-80% [4]. Also related to other diseases in immunosuppressed individuals, including chronic active EBV, fatal infectious mononucleosis (IM) and EBV-associated haemophagocytic syndrome (EBVAHS) [5-7], EBV is linked to several malignancies such as nasopharyngeal carcinoma (NPC) and Burkitt's lymphoma (BL) [5]. In HIV-infected individuals, EBV is associated with diseases such as oral hairy leukoplakia and AIDS-related non-Hodgkin's lymphoma [5,8].

Though sometimes detectable in the immunocompetent [9], EBV DNA is found in greater concentrations in immunosuppressed populations [10-13]. The presence of circulating EBV DNA does not always correlate with symptomatic infection, nor does it predict clinical disease in immunocompetent or immunosuppressed individuals [2,9]. Nevertheless, although the correlation between EBV burden and disease status is incompletely understood, several studies have shown an association between symptomatic infection and elevated DNA loads in clinical samples [14,15]. Increasing virus burden is also believed to be a rapid indicator of immunopathological changes preceding and/or underlying the B-lymphocyte driven changes caused by EBV [16]. Therefore, determining EBV DNA loads in EBV-related disorders in immunocompromised populations is an important step towards disease diagnosis, management and treatment [17].

Several methods for quantifying absolute DNA load have been developed since its first application to EBV diagnostics in 1999 [18-20]. These include semi-quantitative, quantitative competitive and real-time PCR methods [21], with each using different means for amplicon detection; visualisation on agarose gel, Southern blot analysis and enzyme immunoassay [21]. Real-time PCR quantification is generally preferred for its wider dynamic range, speed, ease of handling, sensitivity and specificity [2,22-25]. Although commercial assays incorporating probe-based chemistries are available [26,27], in-house methods employing high saturating dyes such as SYBR Green I are more cost-effective and just as sensitive as the widely used TaqMan PCR [21,28-30].

Here, in an effort to ascertain the relationship between EBV DNA load and disease, two real-time quantitative PCR (QPCR) assays using SYBR Green I dye and a single quantification standard incorporating two separate EBV genes, Epstein-Barr nuclear antigen-1 (EBNA-1) and BamHI fragment H rightward open reading frame-1 (BHRF-1), were developed. EBV DNA was measured in a range of clinical samples, including unfractionated whole blood, plasma, PBMC and CSF from patients with EBV-associated disorders or immune dysfunctions. EBV sero-status was also determined for individuals in a population sample to assess the correlation between DNA load and antibody titres.

## Methods

### Groups with EBV-associated diseases or immune disorders

A total of 60 clinical samples from 25 individuals with various EBV-associated diseases or immune disorders were collected between February 2007 and September 2008. Specimen types included EDTA whole blood, plasma, PBMC and CSF. Each patient was assigned a letter (A to Y) and classified into one of four groups. Group 1 consisted of five patients with EBV-related PTLD following matched-unrelated donor haematopoietic stem cell transplantation, generating 40 samples: whole blood (n = 20), plasma (n = 18) and CSF (n = 2). Group 2 consisted of seven patients with IM, with plasma (n = 4) or whole blood (n = 3) samples and Group 3 was based on a single patient with EBVAHS from whom a whole blood sample was available. Group 4 consisted of PBMC (n = 3) and plasma (n = 9) samples from 12 HIV-infected individuals with HIV RNA plasma loads greater than 10,000 copies/ml.

### Population sample

A fifth group was comprised of 218 individuals from a population sample for whom whole blood and serum were collected between 2004 and 2007. This included 46 males and 172 females with a mean age of 39 (SD = 10) and 40 (SD = 9.5) years respectively. These individuals resided in one of four regions in eastern Australia including Brisbane (n = 78), Newcastle (n = 28), Geelong and the western districts of Victoria (n = 45) and Tasmania (n = 67) [31].

### Serology testing

#### EBV-specific antibody detection in the population sample

Quantitative EBV-specific serology was performed on sera from individuals in Group 5 only. EBV VCA IgG antibodies titres were determined by an immunofluorescence assay (IFA) using FITC conjugated anti-human IgG prepared in goats (Sigma-Aldrich, Castle Hill, NSW, Australia). Cells from the B95-8 marmoset cell line productively infected with EBV were grown in 27 mls of RPMI 1640-modified (ThermoFisher Scientific, Scoresby, VIC, Australia) +10% foetal calf serum (FCS) (ThermoFisher Scientific, Scoresby, VIC, Australia) medium containing 3 mls of 0.4 mM phosphonoacetic acid (Sigma-Aldrich, Castle Hill, NSW, Australia). Cells were spotted on 10 well slides (Pathech, Preston, VIC, Australia) and used as the antigen. Four-fold dilutions of known EBV positive sera were used as controls. Samples were diluted using phosphate buffered saline containing 10% FCS four-fold from 1:10 to an endpoint; samples with a titre < 1:10 were reported as negative, whilst titres equal to or greater than 1:10 were defined as positive.

### Molecular testing

#### EBV gene targets, beta-globin and PCR controls

To maximise detection rates and reduce false negative results, two primer sets targeting the highly conserved EBV regions, EBNA-1 and BHRF-1, were used for PCR amplification (Table 1). EBNA-1 is a latent protein required for replication and genome maintenance and is the only viral protein consistently expressed in EBV-infected cells [32,33]. BHRF-1 is expressed in lytic infection and confers anti-apoptotic properties similar to Bcl-2 for enhancing cell survival [34]. Groups 2-5 were evaluated by both PCR targets, while inadequate sample volume limited testing to EBNA-1 in Group 1. The beta-globin gene targeting the TAL57 region was used as a 'house-keeping' gene to control for PCR inhibitors and check for DNA integrity [35]. All samples were subjected to beta-globin PCR prior to EBV QPCR. Contamination was monitored by the use of PCR-grade water and no template DNA controls.

**Table 1:** Oligonucleotides used for EBV QPCR, beta-globin detection, construction of plasmid and PCR thermal cycling conditions.

Target	Primer Name	Oligonucleotide Sequence 5'-3'	Amplicon Length	GenBank Accession (position)	Reference	Optimised PCR Thermal Cycling Conditions
**EBNA-1**	QP1	GCC GGT GTG TTC GTA TAT GG	213 bp	AJ507799 (97174-97386)	Stevens et al, 1999	95°C initial denaturation for 10 mins; 58°C annealing
	QP2	CAA AAC CTC AGC AAA TAT ATG AG				
**BHRF-1**	EA-1F	GGA GAT ACT GTT AGC CCT G	208 bp	AJ507799 (42105-42312)	Custom	98°C initial denaturation for 13 mins; 60°C annealing
	EA-2R	GTG TGT TAT AAA TCT GTT CCA AG				
**Plasmid construct (randomised primers in bold)**	EA-F	**CTA TAT GTC TGC TTA CTC CGG CG **/G GAG ATA CTG TTA GCC CTG	554 bp	N/A	Custom	95°C initial denaturation for 10 mins; 55°C annealing
	EB-R	**CGC CGG AGT AAG CAG ACA TAT AG **/CAA AAC CTC AGC AAA TAT ATG AG				95°C initial denaturation for 10 mins; 55°C annealing
**Beta-Globin TAL57**	BG-1F	TAG CAA CCT CAA ACA GAC ACC A	247 bp	EU760960 (171-417)	Custom	95°C initial denaturation for 10 mins; 61°C annealing
	BG-1R	CAG CCT AAG GGT GGG AAA AT				

Abbreviations: EBNA-1, Epstein-Barr virus nuclear antigen-1; BHRF-1, BamHI fragment H rightward open reading frame-1; mins, minutes

#### DNA extraction and molecular assay design

DNA was isolated from 200 μl of EDTA whole blood, plasma or CSF using the GenElute™ Mammalian Genomic DNA Miniprep Kit^® ^(Sigma-Aldrich, Caste Hill, NSW Australia) according to the manufacturer's instructions, and eluted in 200 μl elution buffer. The QIAamp DNA mini kit (Qiagen, Doncaster, VIC, Australia) was used to extract DNA from PBMC in accordance with the manufacturer's instructions. Extracts were aliquoted in single use volumes to prevent freeze-thaw cycles and stored at -80°C prior to testing. Each reaction mixture was contained in a PCR-certified colourless 200 μl flat capped tube (Integrated Sciences, Willoughby, NSW, Australia) to a final 25 μl volume, comprising of 2.0 μl LightCycler^® ^FastStart DNA Master SYBR Green 1 dye (Roche Diagnostics, Castle Hill, NSW, Australia) at 10× concentration pre-combined with the LightCycler^® ^FastStart enzyme, 0.5 μl of 0.2 mM sense and antisense primers (Invitrogen, Mount Waverley, VIC, Australia), 0.8 μl of 25 mM MgCl_2 _and 5 μl of the DNA eluate. Samples were tested on the 36-well rotor on the Rotor-Gene 6000^® ^analyser (Qiagen, Doncaster, VIC, Australia). PCR was divided into two cycles: a first cycle with three repeats at 40 seconds for each stage, and a second cycle with 40 repeats at 30 seconds per stage. Thermal cycling conditions included an optimised initial denaturation step followed by 95°C denaturation, optimised annealing temperatures and extension at 72°C (Table 1). To ensure complete product formation, a final extension step at 72°C for 5 minutes concluded the PCR. A melt analysis immediately followed at between 60°C to 99°C as a check for amplicon purity. For confirmation, EBNA-1 and BHRF-1 products were electrophoresed in 2% agarose gel containing 1:20 dilution of SYBR^® ^safe DNA gel stain in 0.5× TBE buffer (Invitrogen, Mount Waverley, VIC, Australia).

#### Cloning of EBNA-1 and BHRF-1 DNA targets into plasmid vector pGEM and standard curve construction

**Figure 1:**
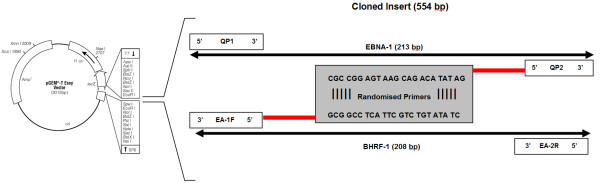
**Plasmid vector pGEM showing location of cloned insert**.

A novel feature of the assay was the design of a quantification standard incorporating both EBNA-1 and BHRF-1 DNA targets in a single plasmid (Figure 1). This was done to minimise the necessity for two separate EBV standards, thus reducing costs and labour. The EBNA-1 and BHRF-1 DNA targets were linked using randomised primers (Table 1) and inserted into the pGEM vector, using the pGEM^®^-T Easy Vector System II (Promega Corporation, Alexandria, NSW, Australia) according to the manufacturer's instructions. The cloned targets were then purified using the PureYield™ Plasmid MidiPrep System (Promega Corporation, Alexandria, NSW, Australia), and stored in single use aliquots. Target copy number was calculated following double stranded DNA approximation using the Beckman DU^® ^530 Life Science UV/Vis spectrophotometer (Beckman Coulter, Gladesville, NSW, Australia). A new plasmid aliquot was used for standard curve dilution for each PCR run consisting of three replicates starting at 10^1 ^to 10^6 ^copies/5 μl. PCR runs were accepted when the standard curve correlation co-efficient was ≥ 0.99.

#### Product identification, reproducibility, sensitivity, limit of detection and specificity

PCR products were identified by an amplification curve, melt analyses and amplification efficiency generated by the Rotor-Gene™ 6000 Software 1.7 (Build 90). Positive EBV DNA samples had a cycle threshold (CT) less than 40, and melted between 86°C to 87°C with an average amplification efficiency of 1.74. PCR products for EBNA-1 DNA and BHRF-1 DNA were identified on agarose gel by 213 bp and 208 bp bands, respectively. Reproducibility studies consisting of triplicates of each standard curve dilution (10^1^-10^5 ^copies/5 μl) were performed prior to testing. Intra-assay variation was determined in three repeat assays tested within 24 hours on three consecutive days. Inter-assay variation was assessed using three different batches of the same PCR master mix kit. Sensitivity was determined by end-point PCR using gel electrophoresis. To establish the minimum DNA copy number that could be reliably detected, ten plasmid replicates spanning 10^0 ^to 10^2 ^copies/5 μl were assayed in three separate runs. Primer specificity was verified on the Basic Local Alignment Search Tool on GenBank and by assaying known cytomegalovirus (CMV), human herpesvirus 6 (HHV6), HIV and varicella zoster (VZV) positive samples. The EBV QPCR was evaluated against an external quality assurance program (Quality Control for Medical Diagnostics (QCMD), Glasgow, Scotland, http://www.qcmd.org/ for EBV QPCR in 2008 and 2009.

#### Viral load calculation and result interpretation

Viral load calculations were based on DNA extraction volume and final elution volumes as well as the number of replicates tested. Samples were extracted and eluted in equal quantities, keeping ratios constant. Hence, the amount of sample used for PCR (5 μl) was multiplied by a factor of 200 (elution volume) and divided by the number of replicates to obtain a final measurement expressed as DNA copies per millilitre (copies/ml) of sample. This unit of measurement has close correlations with copies per microgram of DNA, therefore does not require normalisation to the amount of input DNA [36]. Furthermore, copies/ml removes unnecessary processing steps and reduces costs, as well as minimising sample volume for testing. EBV DNA was quantifiable in a dynamic range spanning six logarithms with the minimum reportable viral load at 2.0 × 10^2 ^copies/ml of sample. Samples with no detectable target DNA were assigned a load of zero and resulted as negative.

### Statistical calculations

Data analysis was conducted with SPSS version 17. Spearman's (rho) correlation co-efficient was used to assess the correlations between EBNA-1 and BHRF-1 DNA loads and VCA IgG antibody titres.

## Results

### Performance of EBV QPCR assays: reproducibility, sensitivity, detection limit and specificity

The intra-assay and inter-assay co-efficient of variation for EBNA-1 and BHRF-1 QPCRs are shown in Table 2. Both EBV targets were detected at levels as low as 2.0 × 10^2 ^copies/ml of sample. However, the reliable limit of detection for both EBNA-1 and BHRF-1 DNA was 2.0 × 10^3 ^copies/ml, where the proportion that were detected (positivity ratio) were 97% and 93% respectively. Primers showed no cross reactivity to other herpesviruses (data not shown). All samples in both the 2008 and 2009 QCMD programs were correctly identified using the EBNA-1 primers.

**Table 2:** Intra- and inter-assay co-efficient of variation for EBNA-1 and BHRF-1 QPCRs.

DNA Target (copies/5 ul)	Mean CT	Mean R-G 6000™ Results (copies/5ul)	Standard Deviation of R-G 6000™ Results (copies/5ul)	Mean % Variation	COV (%)	Mean R^2^
**EBNA-1 Intra-Assay Variation (same day)**						

100,000	18.03	87,329	6,670	12.68%	7.64	0.991
10,000	21.28	11,735	3,092	26.30%	26.34	
1,000	25.21	1,057	100	7.00%	9.50	
100	29.10	103	38	32.12%	37.25	
10	32.91	11	6	46.16%	57.50	

**EBNA-1 Intra-Assay Variation (different days)**						

100,000	16.94	89,643	8,164	11.00%	9.11	0.998
10,000	20.31	10,678	1,207	10.00%	11.31	
1,000	23.85	1,133	129	16.00%	11.41	
100	27.65	102	4	3.00%	3.90	
10	31.42	10	2	17.88%	18.95	

**BHRF-1 Intra-Assay Variation (same day)**						

100,000	17.23	97,884	9,144	8.08%	9.34	0.994
10,000	20.91	9,852	542	4.45%	5.50	
1,000	24.38	1,146	202	16.12%	17.64	
100	28.43	94	19	17.70%	20.51	
10	31.95	11	5	35.38%	41.91	

**BHRF-1 Intra-Assay Variation (different days)**						

100,000	18.05	105,387	4,621	6.02%	4.38	0.997
10,000	21.75	9,779	818	6.23%	8.37	
1,000	25.23	1,042	141	11.63%	13.55	
100	29.06	89	18	13.76%	19.88	
10	32.66	12	2	25.30	16.53	

**EBNA-1 Inter-Assay Variation**						

100,000	19.87	101,644	14,058	10.99%	28.35	0.990
10,000	23.75	10,660	1,471	13.65%	13.80	
1,000	27.68	1,084	191	18.67%	17.60	
100	31.71	111	49	33.58%	43.75	
10	35.86	12	9	65.03%	75.85	

**BHRF-1 Inter-Assay Variation**						

100,000	17.30	109,065	14,266	10.01%	13.08	0.990
10,000	21.49	9,209	2,154	16.84%	23.39	
1,000	25.49	860	251	22.19%	29.15	
100	29.01	108	49	35.53%	45.33	
10	32.29	15	8	69.41%	57.23	

Abbreviations: CT, cycle threshold; Mean % variation, average percentage variation between the calculated (Rotor-Gene results) and the given concentration (DNA target); COV, co-efficient of variation is the ratio of standard deviation to the mean; R^2^-value, square root of the correlation co-efficient - in quantitation PCR describes the percentage of the data which matches the hypothesis that the standards conform to a line of best fit.

### EBV detection and load in EBV-associated disease states and immunocompromised individuals

Of the 60 samples from 25 immunocompromised patients, 30 (50%) samples from 16 (64%) patients had quantifiable viral load using one or other of the EBV DNA targets, EBNA-1 or BHRF-1 (Table 3). EBV DNA was detected in 100%, 85.7%, 100% and 33.3% of patients with PTLD, IM, EBVAHS and HIV-infected individuals (Groups 1 to 4), respectively. EBV DNA loads were detectable at ranges from 2.0 × 10^2 ^to 1.3 × 10^8 ^copies/ml in these clinical samples, with the highest EBV DNA load recorded in an individual with PTLD (1.3 × 10^8 ^copies/ml of sample). High levels were also seen in individuals with IM (2.0 × 10^5 ^copies/ml of sample), EBVAHS (1.1 × 10^5 ^copies/ml whole blood), and HIV infection (5.6 × 10^3 ^copies/ml of sample).

**Table 3:** EBV DNA loads in various EBV-associated disease states and immunocompromised individuals.

Group	Patient ID	Sex/Age	Condition	Specimen (Positive/n Tested)	Target	Detectable EBV DNA Load (copies/ml)	Clinical Notes
1.	A.	M/46y	PTLD	Plasma (5/6)	EBNA-1	Day +32 - 8.0 × 10^2^	MUD HSCT for AML; EBV VCA IgG positive pre-Tx; Plasma collected on Days +32, +39, +46, +60, +75 and +81 for EBV QPCR; Plasma EBV (qualitative) PCR positive on Days +75, +78 and +81; Treatment with Foscarnet and Rituximab after Day +75; Died of pneumonia on Day +88
						Day +46 - 1.0 × 10^3^	
						Day +60 - 8.8 × 10^3^	
						Day +75 - 1.1 × 10^6^	
						Day +81 - 2.3 × 10^5^	
				CSF (2/2)	EBNA-1	Day +75 - 1.3 × 10^6^	CSF collected on Days **+75 **and **+78**
						Day +78 - 2.7 × 10^6^	
	B.	M/42y	PTLD	Whole Blood (1/5)	EBNA-1	Day +95 - 2.0 × 10^7^	MUD HSCT for AML; Plasma EBV (qualitative) PCR positive Day +96; Plasma collected on Day **+95 **for EBV QPCR; Died on Day +99 due to multi-organ failure
	C.	F/59y	PTLD	Plasma (3/6)	EBNA-1	Day +45 - 2.2 × 10^5^	MUD HSCT for AML; CMV reactivation on Day +44, Treatment with Foscarnet and ganciclovir on Day +52; Plasma collected Days +38, +40, **+45**, **+52 **and **+59**; Died on Day +66; EBV VCA IgG positive, HHV6 IgG positive and CMV IgG positive pre-Tx
						Day +52 - 9.6 × 10^3^	
						Day +59 - 3.0 × 10^5^	
				Whole Blood (1/8)	EBNA-1	Day +46 - 6.6 × 10^4^	EDTA collected Days +3, +5, +10, +17, +26, +31, +33, **+46**
	D.	M/48y	PTLD	Plasma (4/6)	EBNA-1	Day +40 - 3.4 × 10^3^	MUD HSCT for AML; EBV VCA IgG positive pre-Tx; Plasma collected Days +28, +33, **+40**, **+47**, **+54**, **+61**; Plasma EBV (qualitative) PCR negative on Day +62; Died Day +72 of multi-organ failure
						Day +47 - 3.6 × 10^4^	
						Day +54 - 3.4 × 10^6^	
						Day +61 - 6.3 × 10^6^	
				Whole Blood (2/2)	EBNA-1	Day +62 - 1.3 × 10^8^	EDTA collected Days **+62 **and **+63**.
						Day +63 - 1.8 × 10^7^	
	E.	F/57y	PTLD	Whole Blood (1/5)	EBNA-1	9.5 × 10^4^	No serology results available however clinical notes indicate EBV reactivation; Plasma EBV (qualitative) PCR positive 9-16 days after VL testing done; negative at 1-7 months thereafter.
**2**.	F.	Unknown	IM	Plasma (1/1)	EBNA-1	3.7 × 10^4^	EBV VCA IgM positive
					BHRF-1	1.6 × 10^4^	
	G.	Unknown	IM	Plasma (0/1)	EBNA-1	0	EBV VCA IgM positive
					BHRF-1	0	
	H.	Unknown	IM	Plasma (1/1)	EBNA-1	7.6 × 10^3^	EBV VCA IgM positive
					BHRF-1	1.5 × 10^3^	
	I.	Unknown	IM	Plasma (1/1)	EBNA-1	2.3 × 10^3^	EBV VCA IgM positive
					BHRF-1	8.7 × 10^4^	
	J.	M/17y	IM	Whole Blood (1/1)	EBNA-1	1.0 × 10^5^	EBV VCA IgM positive
					BHRF-1	1.8 × 10^3^	
	K.	F/19y	IM	Whole Blood (1/1)	EBNA-1	2.2 10^3^	EBV VCA IgM positive
					BHRF-1	5.6 × 10^4^	
	L.	F/53y	IM	Whole Blood (1/1)	EBNA-1	2.0 × 10^5^	EBV VCA IgM positive; acute glandular fever
					BHRF-1	1.8 × 10^4^	
**3**.	M.	M/36y	EBVAHS	Whole Blood (1/1)	EBNA-1	7.5 × 10^4^	EBV (qualitative) PCR positive; died of EBVAHS
					BHRF-1	1.1 × 10^5^	
**4**.	N.	Unknown	HIV	Plasma (1/1)	EBNA-1	0	HIV plasma VL 324, 000 RNA copies/ml
					BHRF-1	1.0 × 10^3^	
	O.	Unknown	HIV	Plasma (0/1)	EBNA-1,	0	HIV plasma VL 13, 000 RNA copies/ml
					BHRF-1	0	
	P.	Unknown	HIV	Plasma (0/1)	EBNA-1,	0	HIV plasma VL 26, 800 RNA copies/ml
					BHRF-1	0	
	Q.	Unknown	HIV	Plasma (0/1)	EBNA-1,	0	HIV plasma VL 21, 300 RNA copies/ml
					BHRF-1	0	
	R.	Unknown	HIV	Plasma (0/1)	EBNA-1,	0	HIV plasma VL 12, 700 RNA copies/ml
					BHRF-1	0	
	S.	Unknown	HIV	Plasma (0/1)	EBNA-1,	0	HIV plasma VL 1, 040, 000 RNA copies/ml
					BHRF-1	0	
	T.	Unknown	HIV	Plasma (0/1)	EBNA-1,	0	HIV plasma VL 17, 700 RNA copies/ml
					BHRF-1	0	
	U.	Unknown	HIV	Plasma (0/1)	EBNA-1,	0	HIV plasma VL 47, 500 RNA copies/ml
					BHRF-1	0	
	V.	Unknown	HIV	Plasma (1/1)	EBNA-1	5.6 × 10^3^	HIV plasma VL 16, 400 RNA copies/ml
					BHRF-1	3.0 × 10^3^	
	W.	Unknown	HIV	PBMC (1/1)	EBNA-1	< 2.0 × 10^2^	HIV PBMC VL 12, 800 RNA copies/ml
					BHRF-1	< 2.0 × 10^2^	
	X.	Unknown	HIV	PBMC (1/1)	EBNA-1	< 2.0 × 10^2^	HIV PBMC VL 12, 700 RNA copies/ml
					BHRF-1	0	
	Y.	Unknown	HIV	PBMC (0/1)	EBNA-1,	0	HIV PBMC VL 118, 000 RNA copies/ml
					BHRF-1		

Abbreviations: Y, years; Group 1 (PTLD), post-transplant lymphoproliferative disease; Group 2 (IM), infectious mononucleosis; Group 3 (EBVAHS), Epstein-Barr virus associated-haemophagocytic syndrome; Group 4 (HIV infection), human immunodeficiency virus; EDTA, ethylenediaminetetraacetic acid; CSF, cerebrospinal fluid; PBMC, peripheral blood mononuclear cells; EBNA-1, Epstein-Barr virus nuclear antigen-1; BHRF-1, BamHI fragment H rightward open reading frame-1; ml, millilitres; Bold lettering indicates Day QPCR positive post-transplant; AML, acute myeloid leukaemia; MUD; matched unrelated donor; HSCT, haematopoietic stem cell transplantation; CMV, cytomegalovirus; VCA, viral capsid antigen; Ig, immunoglobulins; EA-D, early antigen-diffuse; EA-R, early antigen-restricted; VL, viral load

In Group 1 (PTLD), EBV DNA concentrations spanned six logarithms and were detected in multiple samples from early to end-stage disease. EBV DNA loads increased sequentially following transplantation, decreased after anti-viral therapy in Patients A and C and peaked ten days prior to death in Patients A to D. EBV DNA loads were detectable in some samples, but were absent in others. In Patient D, plasma EBV DNA was qualitative PCR negative on Day +62 whilst simultaneously QPCR positive in whole blood. EBV-specific serology results were available for four patients, and confirmed EBV infection prior to the transplant. Four patients died as a result of PTLD complications, on average +81.25 days post transplantation. In Group 2 (IM), EBV DNA was quantifiable from 1.5 × 10^3 ^to 2.0 × 10^5 ^copies/ml. One sample was negative for EBV DNA (Patient G), despite a positive EBV VCA IgM profile. Group 3 (EBVAHS) EBV DNA load results were similar to Group 2, however Patient M died as a consequence of the disease condition. In Group 4 (HIV), EBV DNA was detectable in both plasma and PBMC ranging from 2.0 × 10^2 ^to 5.6 × 10^3 ^copies/ml. However, 50% of these samples were below 2.0 × 10^3 ^copies/ml.

### EBV detection and load in the population sample

EBNA-1 and BHRF-1 DNA were detected in 11.0% and 21.6% of Group 5 (the population sample), respectively; 22.5% of samples were positive for at least one EBV DNA target (Table 4). Of the 24 EBNA-1 DNA positive samples, 91.7% were also BHRF-1 DNA positive, and of the 47 BHRF-1 DNA positive samples, 46.8% were also EBNA-1 DNA positive. Viral loads (combined targets) were detectable between 2.0 × 10^2 ^to 6.2 × 10^4 ^copies/ml of whole blood, but 54.2% and 85.1% of samples were below 2.0 × 10^3 ^copies/ml for EBNA-1 and BHRF-1 DNA levels, respectively. All samples with measurable EBV DNA were EBV VCA IgG antibody positive, which were found in 95.9% of the population sample. There was a modest correlation between VCA IgG antibody titres and BHRF-1 DNA load (Spearman's rho = 0.13, p = 0.05) and a weaker (not statistically significant) correlation between EBNA-1 DNA load and VCA IgG antibody titres (Spearman's rho = 0.11, p = 0.11) (Table 4).

**Table 4:** EBV DNA load and antibody titre detection rates in the population samples (Group 5, n = 218).

Target	Positive n (%)	Detectable Range	Spearman correlation (p)			
			**EBNA-1 DNA load**	**BHRF-1 DNA load**	**Combined EBV Targets DNA load**	**VCA IgG**

EBV EBNA-1 DNA load (copies/ml)	24 (11.0%)	2.0 × 10^2 ^- 9.1 × 10^4^	1.00			
EBV BHRF-1 DNA load (copies/ml)	47 (21.6%)	2.0 × 10^2 ^- 3.3 × 10^4^	0.63 p < 0.001	1.00		
Combined EBV targets DNA load (mean of BHRF & EBNA loads where both positive) (copies/ml)	49 (22.5%)	2.0 × 10^2 ^- 6.2 × 10^4^	0.73 p < 0.001	0.97 p < 0.001	1.00	
Viral capsid antigen IgG (titres)	209 (95.9%)	1:10 - 1:5120	0.11 p = 0.11	0.13 p = 0.05	0.14 p = 0.04	1.00

Abbreviations: EBV, Epstein-barr virus; EBNA-1, Epstein-barr virus nuclear antigen-1; BHRF-1, BamHI fragment H rightward open reading frame-1; VCA, viral capsid antigen; IgG, immunoglobulin G; Pos, positive

## Discussion

With increasing availability of nucleic acid testing (NAT) methods, measuring EBV DNA in blood has proven valuable in diagnosing and monitoring PTLD [16,21,22,37-41], NPC [42,43], IM [13,44], EBV infection in HIV-infected individuals [8,13,45], BL [13] and chronic active EBV infection [18,46]. In this study, we successfully developed two in-house QPCR methods incorporating a novel single quantification standard containing two EBV DNA targets for measuring viral load on the Rotor-Gene 6000™. Substituting SYBR Green I dye as a fluorescent marker for product accumulation over fluorogenic probes, this method proved useful for quantifying EBV DNA concentrations in clinical samples from individuals with a variety of EBV-associated disorders or immune dysfunctions and in a healthy population sample.

Previous studies in PTLD have found that EBV DNA loads increased with disease progression and decreased with remission of lymphoproliferation [47,48]. This pattern was observed in Group 1, where EBV DNA loads appeared to be correlated with disease status. We found similar EBV DNA loads to those previously reported, with most studies showing EBV DNA concentrations ranging from 5.0 × 10^2 ^to 2.0 × 10^7 ^copies/ml in whole blood, plasma and serum [37,49,50]. EBV DNA was also detected in CSF at concentrations comparable to plasma, however detectable CSF EBV DNA has been previously reported only in association with acquired immunodeficiency syndrome (AIDS)-related brain lymphoma [51]. The significance of EBV DNA in CSF of PTLD remains to be elucidated.

EBV DNA loads in IM patients were also similar to those reported in the literature [13,22,26,44,52], although some authors described loads as high as 10^6 ^and 10^7 ^copies/ml [12,46,53]. In Group 3, EBV DNA loads were consistent with acute phase EBVAHS [46,54], and correlated with the deterioration of the patient's disease condition. Elazary et al also found that a viral load ranging from 10^4^-10^5 ^copies/ml was associated with poor patient outcome [54]. One study found much higher EBV DNA loads (up to 10^7 ^copies/ml) [55], but this may have been due to differences in sample type and detection methods. In Group 4, EBV DNA was detected in 33% of samples (22% of plasma, 67% of PBMC), compared to 34% to 76% positivity reported in other studies [8,26]. Notably however, these studies used whole blood for quantifying EBV DNA load, which could have increased the probability of viral DNA detection. As none of the Group 4 patients were known to have EBV-related disease, low positivity ratios and viral loads were expected.

Similar to our findings, the literature describes EBV DNA detectable from 10^2 ^to 10^4 ^copies/ml and positivity ratios up to 29% in whole blood of healthy individuals [11-13,26,38,56-59]. However, DNA loads as high as 5.5 × 10^5 ^copies/ml of whole blood and a positivity ratio of 72% have been reported [58]. Differences in the results may be attributable to more sensitive methods associated with nested PCR and dual-labelled probes [58]. Interestingly, another study showed 100% EBV DNA positivity in whole blood, although DNA loads were all below the detection limit of the assay (2.0 × 10^3 ^copies/ml) [38].

In the population sample the EBV VCA IgG antibody detection rate was consistent with levels of EBV sero-positivity in Western societies [2]. One study previously showed a correlation between EBV VCA IgG antibody titres and EBV viral load (detectable versus non-detectable) [60]. We similarly found a modest correlation with quantitative BHRF-1 DNA loads, and a weaker (not statistically significant) correlation with EBNA-1 DNA load (see Table 4).

We noted some discrepancies in our measures of EBV positivity. In one PTLD patient (Patient D), plasma was qualitative EBV PCR negative whilst simultaneously reporting an EBV DNA load of 1.3 × 10^8 ^copies/ml in whole blood. However, a growing number of studies have shown that cell-associated EBV is detectable before plasma EBV DNA and can persist without accompanying plasma DNA loads [21,48]. In Group 2, Patient G, despite being EBV VCA IgM antibody positive, was EBV QPCR negative. As EBV DNA loads can change rapidly from being undetectable to being very high in a short period of time [38], it is possible that sampling occurred late in the convalescent phase where low EBV DNA positivity ratios of 44% have been previously reported [46]. Other factors contributing to DNA load variation include differences in sample type, method of extraction or NAT, and target chosen for PCR amplification.

As specimen type is known to influence DNA loads and impact on assay performance [36], unfractionated EDTA whole blood was used for DNA quantification where possible. The dynamic changes of EBV DNA are better reflected in circulating whole blood [38], which also contains all the compartments that may harbour virus [13,21,61]. However, despite reports of greater test sensitivity with whole blood [12,36], EBV DNA load has also been quantified in PBMC [14,16,62-64]. Although infection is typically associated with cell compartments [8,12,13], EBV DNA is also found in cell-free blood partitions such as plasma or serum, usually in fragmented, cell-derived form [12]. In this study, 2 of 9 plasma samples from HIV-infected patients had detectable EBV DNA, compared to 2 out of 3 PBMC samples. As we did not have simultaneous plasma and PBMC samples from the same individuals, we were unable to assess the differences in viral load between these compartments. Further studies comparing suitability of different sample types in various EBV-related diseases and immune disorders are required.

The method of DNA purification is known to affect viral load measurements. One study showed yield from manually extracted DNA was 57% higher than that of robotic systems [65]. Therefore, to improve DNA recovery and maximise PCR sensitivity, samples here were purified using a commercial silica-based column method [61,66]. For optimal quantitation results, an earlier study showed that DNA should be subjected to PCR within one to two weeks post-extraction [67]. Here, delay between extraction and testing could have contributed to low DNA loads and positivity ratios in clinical samples. Furthermore, DNA from blood samples that had undergone more than four freeze-thaw cycles were found to be partially degraded [68]. Since the clinical samples used here were tested retrospectively, monitoring these conditions were not possible.

EBV DNA loads also vary according to type and size of gene target [69]. Ryan *et al*, found assay sensitivity was dependent on the specific gene segment and that different targets had varying lower limits of detection [15]. For EBV, BamHI-W is reportedly 10 times more sensitive than other targets for PCR, allowing for detection of viral DNA at trace amounts [8,13,15]. However, precise quantification of viral genomes is complicated by the number of reiterated BamHI-W sequences among EBV strains, which typically ranges between 7 and 11 repeats per genome [15]. To avoid overestimation in this study, we chose to use the next most sensitive EBV gene; EBNA-1 [15], and an abundantly expressed gene, BHRF-1, for QPCR.

Despite targeting highly conserved EBV regions, selective drop out of amplifiable EBV DNA at the EBNA-1 and BHRF-1 loci was observed in Group 4 (Patients N and X), and in 25 of 218 (11.5%) whole bloods from the population sample. Instead of amplifying both EBV DNA genes, only one target was detected, 93% of which had viral loads less than 2.0 × 10^3 ^copies/ml. As beta-globin was detected in all samples, PCR inhibitors and/or defective nucleic acid purification methods were excluded [70]. Alternatively, selective drop out may have been due to low viral load and/or sampling error [71]. Since load determination is reliant on the amount of EBV genomes pipetted into a reaction and assumes viral homogeneity, QPCR results, particularly at low viral load levels are prone to random sampling error. This phenomenon is well documented in DNA quantification and results in less reliable viral load measurements [70,71]. Therefore, samples reporting low levels of target nucleic acid may not be reproducible in repeated assays from the same or different specimens [72].

Currently, there are no standardised methods for measuring EBV DNA, complicating inter-laboratory comparisons in multicentre studies of EBV-related diseases. Standardisation is difficult as PCR assay conditions vary between laboratories, leading to variations in the accuracy and reproducibility of viral load quantification [21]. Although there appears to be a strong concordance between laboratories for qualitative EBV DNA estimates, there continues to be marked inconsistency in quantitative results [73]. It has been suggested that the use of unfractionated whole blood [26] or an international calibration standard could be the first step towards standardisation [73]. However, instrumentation, chemistries, gene targets and other test-related aspects remain diverse. One solution for enabling inter-laboratory comparisons is the distribution of proficiency panels such as QCMD. Such programs have already been used for assessing methods for the detection and quantification of EBV and other viruses [27,74,75].

## Conclusion

This is the first reported study that uses the SYBR Green I dye on the Rotor-gene 6000™ with a novel quantification standard containing two EBV targets for measuring EBV DNA load. The assays proved successful in the quantification of EBV genomes in clinical cases and should be considered as a cost effective and sensitive PCR alternative to probe-based assays. This approach can be modified to detect and quantify other latent herpesviruses such as HHV6, CMV, and VZV. This procedure is suitable for robotics and automation, and would be a useful addition in larger laboratories.

## Competing interests

The authors declare that they have no competing interests.

## Authors' contributions

MLL developed the assays, carried out all of the DNA work, assisted in the data analysis and result interpretation, and writing of the manuscript. On behalf of the Ausimmune Investigator group, RML supplied the whole blood and serum from the population sample, and was involved in the data analysis. VMR aided in primer design and JT performed the serology testing. MLL, DED, VMR, RML and ALP were involved in the design and conception of the study. All authors have read, reviewed and approved the final manuscript.
